# Optimal wideband digital fractional-order differentiators using gradient based optimizer

**DOI:** 10.7717/peerj-cs.2341

**Published:** 2024-10-14

**Authors:** Mohammed Ali Mohammed Moqbel, Talal Ahmed Ali Ali, Zhu Xiao

**Affiliations:** 1 College of Computer Science and Electronic Engineering, Hunan University, Changsha, China; 2 Shenzhen Research Institute, Hunan University, Shenzhen, China

**Keywords:** Digital fractional order differentiator, Infinite impulse response, Gradient based optimizer

## Abstract

In this paper, we propose a novel optimization approach to designing wideband infinite impulse response (IIR) digital fractional order differentiators (DFODs) with improved accuracy at low frequency bands. In the new method, the objective function is formulated as an optimization problem with two tuning parameters to control the error distribution over frequencies. The gradient based optimizer (GBO) is effectively employed on the proposed objective function. A wide range of design examples are presented to illustrate the effectiveness of the proposed approach. The proposed approximations are compared to those of recent literature in terms magnitude, phase, and group delay errors. The result reveal that our method can attain approximations have a favorable low frequency performance (with about 60% of relative magnitude error reduction) and maintain a comparable accuracy at most of the Nyquist band to those of the existing ones. Thus, our approximations can be attractive for low frequency applications.

## Introduction

Fractional derivative *s*^*ν*^, 0 < *ν* ∈ ℝ < 1, has gradually superseded conventional derivative *s* in many engineering disciplines, *e.g.*, signal processing, control, and biomedical applications (Krishna, 2011; Nayak et al., 2019; Krishna, 2019; Habeb et al., 2024). This indeed owing to the ability of fractional operator to model the dynamic behavior of physical systems more accurately (Xue, 2017). This paper focuses on the implementation of *s*^*ν*^ by means of infinite impulse response (IIR) digital filters (referred to as digital fractional-order differentiators (DFODs)) to compute the fractional order derivatives of discrete-time signals. The frequency response to be approximated is

(1) \begin{eqnarray*}{H}_{fod}(j\omega ,\nu )=(j\omega )^{\nu }{e}^{-j\omega \tau }={\omega }^{\nu }{e}^{j( \frac{\nu \pi }{2} -\omega \tau )},~~~\omega \in {\Omega }_{p},\end{eqnarray*}

where $j=\sqrt{-1}$, *ω* is the angular frequency in radians/s, Ω_*p*_ is the frequency band of interest, and *ν* is a real number in the open interval (0, 1) to define the order of the fractional derivative. This design problem has received a lot of research attention in the past two decades, yet remains an open challenge with much room for accuracy enhancement, particularly for applications with low frequency signals (Visweswaran, Varshney & Gupta, 2011; Rajasekhar & Krishna, 2020; Rajasekhar, 2022; Ali et al., 2024).

The most well-known design method for DFODs is the *discretization* method (Krishna, 2011; El-Khazali, 2014; Xue, 2017), which maps *s*^*ν*^ into the *z*-domain by using an expansion technique (*e.g.*, continued fraction expansion (CFE)) and an *s*-to- *z* transformation g(*z*^−1^), where g(*z*^−1^) ≈ *s* (*e.g.*, Tustin and Al-Alaoui operators). It either directly expands ${ \left( \text{g}({z}^{-1}) \right) }^{\nu }$ using an expansion technique (direct scheme) (Visweswaran, Varshney & Gupta, 2011; Maione, 2013; Rajasekhar & Krishna, 2018; Rajasekhar & Krishna, 2020; Rajasekhar, 2022), or expands *s*^*ν*^ using an expansion technique and then substitutes *s* → g(*z*^−1^) in the resultant *s*-domain expression (indirect scheme) (Krishna, 2011; Ali et al., 2024). Although simple and straightforward, such method does not provide the user flexibility to specify the frequency band of interest. Worse yet, it tends to come up with approximations in limited frequency regions only, as easily entrapped into a local optimum on the error surface.

The above-mentioned drawbacks of the discretization method have motivated many researchers towards the use of optimization techniques to design DFODs that are optimal in some sense (*e.g.*, Nayak et al., 2019; Rana et al., 2016; Barsainya, Rawat & Kumar, 2016; Mahata et al., 2018a; Mahata et al., 2019; Mahata et al., 2018b; Ali et al., 2020). Here, the design problem is formulated as an optimization problem (*e.g.*, to minimize a given magnitude error function) that can be tackled using an iterative procedure. However, in most cases, the formulated optimization problem is highly non-convex and very hard to solve. One way to get around this issue involves approximating the original non-convex problem by a convex one that can be solved using gradient-based techniques (Johansson, 2013; Nongpiur, Shpak & Antoniou, 2014; Lai & Lin, 2016). However, such a way usually requires an appropriate initial solution, and results in sub-optimal solutions as it adopts an approximation of the design problem rather than the problem itself.

As an alternative way, the non-convex problem can be directly tackled using global search algorithms (*e.g.*, heuristic and metaheuristic techniques) that are equipped with stochastic operators to escape from the sub-optimal solutions. For example, least squares DFODs (optimal in the *L*_2_ sense) have been computed using cat swarm optimization (Mahata et al., 2018a), moth flame optimization (Mahata et al., 2019), flower pollination algorithm (Mahata et al., 2018b), ant lion optimization (Nayak et al., 2019), salp swarm algorithm (Nayak et al., 2021), shuffled frog leaping algorithm (Mohan & Rao, 2021), hybridization of grey wolf optimizer with cuckoo search algorithm (Mouhou & Badri, 2022), whale optimization (Nayak & Kumar, 2024), artificial rabbit optimization (Sinha & Kar, 2024), and quantum-inspired evolutionary algorithm (Siddiqui, Mani & Singh, 2024). Whereas, *L*_1_ optimal DFODs that shown to possess flat magnitude response have been proposed using multi-verse optimizer (Ali et al., 2020). In general, metaheuristic techniques have been observed to attain accurate approximations over wide frequency bands in short computation time. However, the attained approximations mostly have a comparatively large error near the band edges, *i.e.,* at *ω* ∈ [0, 0.15*π*] and *ω* ∈ [0.85*π*, *π*]. In such methods, the only way to improve the accuracy at low frequencies in order to meet the requirements in low-frequency applications is to adopt high-order approximations. This, however, increases both the design and implementation complexity of the system, hindering the versatility of such approximations in many applications, such as servo control (Sabatier et al., 2015; Visweswaran, Varshney & Gupta, 2011), QRS detection (5 − 22 Hz (Nayak et al., 2019)), and measurements of seismic event signals (15 − 60 Hz (Mousa, 2020; Li et al., 2024a)), heating rate (<100 Hz (Kruttiventi, Wu & Frankel, 2010; Zhao et al., 2024)), and vibration signals(<500 Hz (Pelegri Sebastia et al., 2009; Li et al., 2024b)).

The motivation of this work lies in the need for a design method that enables considerable reduction of the low-frequency error of approximations without calling for higher filter order. Such approximations can be useful in low-frequency applications, *e.g.*, the above-mentioned applications, as they can fulfill the accuracy requirements with as low order as possible. In previous metaheuristic-based approximations, the large error near band edges can be attributed to two main reasons. The first reason is that previous works often utilize objective functions that imply an equal priority for all frequencies, *e.g.*, uniform *L*_2_-norm and *L*_1_-norm objective functions. Since *H*_*fod*_(*jω*, *ν*) in Eq. (1) cannot be approximated arbitrarily well neither at *ω* = 0 nor at *ω* = *π* using real filter approximations (which have real values at these frequencies), the error near these frequencies tends to be large when uniform objective function is used. This paper focuses on using a weighted objective function to give a higher priority for low frequency bands during the optimization progress. Weighted objective functions have been successfully used in different digital filter design problems. For example, to attain digital differentiators (approximations of conventional differentiator, *H*_*dif*_(*jω*) = *jω*) with high accuracy in the vicinity of *ω* = 0, Ali et al. (2019) have introduced a weighted *L*_1_-norm objective function that has an inverse weight, *i.e.,* the weight is inversely proportional to *H*_*dif*_(*jω*).

The second reason is that, despite the promising search capability of metaheuristic techniques, the global optimal solution of highly complex problems cannot be definitely attained. As is well known, traditional metaheuristic techniques use a population of solutions and a random direction (*i.e.,* without utilizing any gradient guidance) to search for the optimal solution in the problem search space, and they yield different solution for each independent run. For highly non-convex problems, they need a lot of runs with tiresome tuning of their controlling parameters to end up with a near global optimum, due to the frequent stagnation in local optima. This is even getting worse with the size of search space of the problem being tackled (Khan et al., 2020). To alleviate these drawbacks, some authors resort to hybridize a metaheuristic technique with gradient decent mechanism to facilitate the search towards the feasible regions of the large search space while preserving the advantages of the population-based mechanism of the metaheuristic itself (Khan et al., 2020; Ahmadianfar, Bozorg-Haddad & Chu, 2020; Habeb et al., 2024). Despite the great success of deep learning models, which are optimized by traditional gradient descent methods, in various fields, *e.g.*, (Li et al., 2024b; Zhao et al., 2024; Chen et al., 2024; Granado & Alkhaled, 2024; K., Ananth & L., 2024), such a hybrid approach can even be more promising in training of deep neural networks (Khan et al., 2020; Habeb et al., 2024). This motivates us to leverage the hybridization of gradient with metaheuristic mechanisms to cope the challenges of this design problem, *e.g.*, the large error near *ω* = 0, where its performance in the design problem of DFODs has not yet been investigated.

In this paper, we propose a new design method for wideband DFODs with improved accuracy at low frequency bands, which is based on a weighted *L*_1_-norm objective function and the gradient-based optimizer (GBO) (Ahmadianfar, Bozorg-Haddad & Chu, 2020). Unlike the weighted objective function in Ali et al. (2019) that merely has an inverse weight, our objective function allows to control the degree of priority given for low frequency bands by raising the inverse weight to an arbitrary real-valued exponent. Moreover, this objective function is formulated to obtain an approximation whose magnitude follows a scaled magnitude of *H*_*fod*_(*jω*), which necessitates to scale the resultant approximation back to fit the unscaled *H*_*fod*_(*jω*). The scale factor is a user-defined, and it can be tuned to improve the accuracy towards the low frequency regions. The search for the global optimal solution is carried out using GBO, which has been recently developed by integrating the population-based mechanism with a search engine based on the gradient-based Newton’s method to explore the feasible regions in the search space. Since its proposal, GBO has been effectively employed to solve many engineering and science problems, *e.g.*, parameter extraction in photovoltaic models (Ismaeel et al., 2021), unit commitment problem (Said et al., 2022), distribution static VAR compensator sizing and placement in electrical systems (Moustafa et al., 2023), optimal tuning of power system stabilizers for a multi-machine power systems (El-Dabah et al., 2023), load frequency control and automatic voltage regulation in four-area interconnected power systems (Ali et al., 2023).

The main contributions of this paper are outlined as follows.

(i) We propose a weighted *L*_1_-norm objective function equipped with two user-defined parameters (*i.e.,* the exponent of the inverse weight and the scale factor of the ideal magnitude response to be approximated) to enable the designer to control the degree of priority given to the low frequency bands.
(ii) We propose a new efficient method for the design of wideband DFODs with improved accuracy at low frequency regions, which is based on a combination of the proposed objective function and a recently-proposed metaheuristic, GBO, that hybridizes the population-based mechanism and a gradient search engine.
(iii) We present design examples with various design criteria to demonstrate the effectiveness of the proposed method. The performance of the proposed method is also compared with that of widely-known metaheuristic algorithms, real-coded genetic algorithm (RCGA) and particle swarm optimization (PSO).
(iv) We compare the proposed DFODs with their recently-proposed counterparts in terms of frequency response and in an application example, QRS complexes detection.

The remainder of this paper is organized as follows. In ‘Problem formulation’, the design problem of DFODs based on the proposed weighted *L*_1_-norm objective function is formulated. ‘Design of DFODs’ presents the design of DFODs, where ‘Gradient based optimizer (GBO)’ outlines GBO and ‘The proposed design method’ introduces the proposed design method. ‘Simulation results’ provides simulation results for design examples with a comparative study for the proposed method with RCGA and PSO. Comparisons of the proposed DFODs with the existed ones in terms of frequency response and QRS complexes detection are given in ‘Frequency response’ and ‘Application examples’ respectively. The conclusions of this work are then highlighted.

## Problem formulation

The transfer function of an IIR digital filter of order *N* = 2*J* can be expressed in the polar form as given in Nongpiur, Shpak & Antoniou (2014)

(2) \begin{eqnarray*}H(z,\mathbf{q})={H}_{0} \frac{\prod _{i=1}^{J} \left( z-{r}_{ai}^{(1)}{e}^{j{\theta }_{ai}} \right) \left( z-{r}_{ai}^{(2)}{e}^{-j{\theta }_{ai}} \right) }{\prod _{i=1}^{J} \left( z-{r}_{bi}^{(1)}{e}^{j{\theta }_{bi}} \right) \left( z-{r}_{bi}^{(2)}{e}^{-j{\theta }_{bi}} \right) } ,\end{eqnarray*}

where

(3) \begin{eqnarray*}\mathbf{q}=[{H}_{0},{r}_{a1}^{(1)},{r}_{a1}^{(2)},{r}_{b1}^{(1)},{r}_{b1}^{(2)},\ldots ,{r}_{aJ}^{(1)},{r}_{aJ}^{(2)},{r}_{bJ}^{(1)},{r}_{bJ}^{(2)},{\theta }_{a1},{\theta }_{b1},\ldots ,{\theta }_{aJ},{\theta }_{bJ}]^{T}\in \mathbb{R},\end{eqnarray*}

where *H*_0_ ∈ ℝ is a multiplier constant. Note that a transfer function of odd order (*i.e., N* is odd) can be easily obtained by putting *J* = (*N* + 1)/2 and assigning ${r}_{a1}^{(1)}$ and ${r}_{b1}^{(1)}$ to zero. The frequency response of *H*(*z*, **q**) is obtained by substituting *z* = *e*^*jω*^ in Eq. (2), *i.e.,*

\begin{eqnarray*}H({e}^{j\omega },\mathbf{q})={H}_{0} \frac{\prod _{i=1}^{J} \left( {e}^{j\omega }-{r}_{ai}^{(1)}{e}^{j{\theta }_{ai}} \right) \left( {e}^{j\omega }-{r}_{ai}^{(2)}{e}^{-j{\theta }_{ai}} \right) }{\prod _{i=1}^{J} \left( {e}^{j\omega }-{r}_{bi}^{(1)}{e}^{j{\theta }_{bi}} \right) \left( {e}^{j\omega }-{r}_{bi}^{(2)}{e}^{-j{\theta }_{bi}} \right) } . \end{eqnarray*}

The desired filter response is obtained by adjusting the filter coefficients **q**. In this work, we aim to obtain **q** such that *H*(*e*^*jω*^, **q**) approximates *H*_*fod*_(*jω*, *ν*) as close as possible. The complex error function to be minimized is given by

(4) \begin{eqnarray*}e(\omega ,\mathbf{q})=[H({e}^{j\omega },\mathbf{q})-{H}_{fod}(j\omega ,\nu )] \forall \omega \in {\Omega }_{p},\end{eqnarray*}

where Ω_*p*_ denotes the frequency band of interest. To control the magnitude and phase errors independently, elliptic error method has been proposed (Lai & Lin, 2016). Here, the transformed complex error function is given by

(5) \begin{eqnarray*}\overline{e}(\omega ,\mathbf{q})=[H({e}^{j\omega },\mathbf{q}){e}^{-j({\phi }_{0}-\tau \omega )}-{\omega }^{\nu }]=[\overline{H}({e}^{j\omega },\mathbf{q})-{\omega }^{\nu }] \forall \omega \in {\Omega }_{p},\end{eqnarray*}

where ${\phi }_{0}= \frac{\nu \pi }{2} $ and $\overline{H}({e}^{j\omega },\mathbf{q})=H({e}^{j\omega },\mathbf{q}){e}^{-j({\phi }_{0}-\tau \omega )}$. Then, the magnitude and phase errors can be given by

(6) \begin{eqnarray*}{\overline{e}}_{m}(\omega ,\mathbf{q})=\mathfrak{R}\mathfrak{e}\{ \overline{e}(\omega ,\mathbf{q})\} +\mathfrak{I}\mathfrak{m}\{ \overline{e}(\omega ,\mathbf{q})\} /50,  {\overline{e}}_{\phi }(\omega ,\mathbf{q})=\angle \overline{H}({e}^{j\omega },\mathbf{q}).\end{eqnarray*}

### Magnitude response error

Since our method utilizes a metaheuristic technique to seek for the global optimal, we adopt the *L*_*p*_-norm of the non-convex error, *i.e.,*

(7) \begin{eqnarray*}\parallel {{\tilde {e}}_{m}(\omega ,\mathbf{q})}_{p}\parallel = \left( \right. \int \nolimits \nolimits _{0}^{\pi }W(\omega ) \left\vert \right. {|}\overline{H}({e}^{j\omega },\mathbf{q}){|}-{\omega }^{\nu }{ \left\vert \right. }^{p}d\omega { \left( \right. }^{1/p},\end{eqnarray*}

where *W*(*ω*) is a positive weighting function that can be used to control the magnitude error at certain frequencies, *e.g.*, (Ali et al., 2019). To allow control of magnitude error over frequencies, we introduce the following *L*_1_ objective function

(8) \begin{eqnarray*}\parallel {{\tilde {e}}_{m}(\omega ,\overline{\mathbf{q}})}_{1}\parallel =\int \nolimits \nolimits _{0}^{\pi } \frac{ \left\vert \right. {|}{\overline{H}}_{\gamma }({e}^{j\omega },\overline{\mathbf{q}}){|}-{ \left( \omega /\gamma \right) }^{\nu } \left\vert \right. }{{ \left( {\omega }^{\nu } \right) }^{\lambda }+{\epsilon }_{m}} d\omega ,\end{eqnarray*}

where ${\overline{H}}_{\gamma }({e}^{j\omega },\overline{\mathbf{q}})={\gamma }^{-\nu }\overline{H}({e}^{j\omega },\mathbf{q})$, *γ* ≥ 1 and *λ* ≥ 1 are real numbers, and *ϵ*_*m*_ is a small number that included to avoid the division by zero. Thus, our objective is to determine optimal values of $\overline{\mathbf{q}}$ such that ${{\tilde {e}}_{m}(\omega ,\overline{\mathbf{q}})}_{1}$ is minimized. The parameters *γ* and *λ* can be tuned to obtain the required magnitude error profile.

### Phase response error

We adopt the phase error given in Eq. (6) to allow independent minimizing of magnitude and phase errors. That is, our objective is to minimize the error given by

(9) \begin{eqnarray*}{\tilde {e}}_{\phi }(\omega ,\overline{\mathbf{q}})=\angle {\overline{H}}_{\gamma }({e}^{j\omega },\overline{\mathbf{q}})={\tan \nolimits }^{-1} \left\{ \right. \frac{\mathfrak{I}\mathfrak{m}\{ {\overline{H}}_{\gamma }({e}^{j\omega },\overline{\mathbf{q}})\} }{\mathfrak{R}\mathfrak{e}\{ {\overline{H}}_{\gamma }({e}^{j\omega },\overline{\mathbf{q}})\} } \left( \right. , \forall \omega \in {\Omega }_{p}.\end{eqnarray*}

Since tan(*x*) ≈ *x* for small *x*, we approximate ${\tilde {e}}_{\phi }(\omega ,\overline{\mathbf{q}})$ as

(10) \begin{eqnarray*}{\tilde {e}}_{\phi }(\omega ,\overline{\mathbf{q}})\approx \frac{\mathfrak{I}\mathfrak{m} \left\{ \right. {\overline{H}}_{\gamma }({e}^{j\omega },\overline{\mathbf{q}}) \left( \right. }{\mathfrak{R}\mathfrak{e} \left\{ \right. {\overline{H}}_{\gamma }({e}^{j\omega },\overline{\mathbf{q}}) \left( \right. } , \forall \omega \in {\Omega }_{p}.\end{eqnarray*}

### Stability constraints

To make sure of the stability of the digital filter, all the poles should lie inside the unit circle. Here, we derive the constraints to strict the value of the poles with some margin. The stability constraint can be stated as follows:

(11) \begin{eqnarray*}{r}_{b}\leq 1-{\epsilon }_{s}\end{eqnarray*}

where *ϵ*_*s*_ is stability margin.

### Optimization problem

In the optimization problem, we aim at minimizing the magnitude and phase response errors simultaneously. That is, we use a weighted sum of magnitude and phase errors. Then, the optimization problem is formulated as follows

\begin{eqnarray*}\mathrm{minimize}_{\overline{\mathbf{q}}}~~~{\mu }_{m}\parallel {\tilde {e}}_{m}(\omega ,\overline{\mathbf{q}}){\parallel }_{1}+{\mu }_{\phi }\parallel {\tilde {e}}_{\phi }(\omega ,\overline{\mathbf{q}}){\parallel }_{1} \end{eqnarray*}

(12) \begin{eqnarray*}\text{subject to}~~~~{r}_{b}\leq 1-{\epsilon }_{s},\end{eqnarray*}

where *μ*_*m*_ and *μ*_*ϕ*_ are weighting factors (*μ*_*m*_ + *μ*_*ϕ*_ = 1).

## Design of DFODs

In this section, we illustrate the design of wideband DFODs with improved accuracy at low frequencies. The first subsection explains the metaheuristic to solve the optimization problem formulated so far. In the second subsection, the proposed method is illustrated.

### Gradient based optimizer

The gradient based optimizer (GBO) has been recently proposed in Ahmadianfar, Bozorg-Haddad & Chu (2020) by combining the gradient and population-based methods. Here, the Newton’s method is used to specify the search direction to explore the search space. The search is carried out based on a set of vectors and two main operators, namely, gradient search rule and local escaping operator.

#### Initialization

An objective function, restrictions, and a set of decision variables are all part of an optimization problem. GBO have two control parameters. The first parameter controls the transition from the exploration to exploitation. The second parameter is a probability rate. Like any other population-based metaheuristic, GBO has *N*_*p*_ solution (which is called “vector” in this algorithm), each is a *D*-dimensional array. The vectors are given by

\begin{eqnarray*}{X}_{n,d}=[{X}_{n,1},{X}_{n,2},\ldots ,{X}_{n,D}],~~~~~~~n=1,2,\ldots ,{N}_{p},d=1,2,\ldots ,D. \end{eqnarray*}

Each of these vectors is initially generated in a random manner as follows:

(13) \begin{eqnarray*}{X}_{n}={X}_{min}+rand(0,1)\times ({X}_{max}-{X}_{min}),\end{eqnarray*}

where *X*_*min*_ and *X*_*max*_ are the lower and upper limits of *X* respectively, and *rand*(0, 1) is a random number in [0, 1].

#### Gradient search rule

The gradient search rule manipulates vector movement to better search the feasible region and to obtain better positions in the search space. In GBO, the gradient search rule (GSR) is suggested based on the idea of the gradient-based techniques, particularly, Newton’s gradient-based method, with the intention of improving the exploration tendency and promoting the convergence speed. Since optimization problems are often nondifferentiable, a numerical gradient procedure is exploited rather than deriving the objective function directly.

Gradient-based approaches typically start with an estimated starting point and proceed in a direction determined by the gradient to the next position. The one utilized by GBO is expressed as:

(14) \begin{eqnarray*}{x}_{n+1}={x}_{n}- \frac{2\Delta x\times f({x}_{n})}{f({y}_{n}+\Delta x)-f({y}_{n}-\Delta x)} ,\end{eqnarray*}

where

(15) \begin{eqnarray*}{y}_{n}= \frac{[{z}_{n+1}+{x}_{n}]}{2}  \text{and} {z}_{n+1}={x}_{n}- \frac{2\Delta x\times f({x}_{n})}{f({x}_{n}+\Delta x)-f({x}_{n}-\Delta x)} .\end{eqnarray*}

The *GSR*, which is the main core of GBO, is derived based on Eq. (14), with some customizations to enable the population-based search. It is given by

(16) \begin{eqnarray*}GSR=randn\times {\rho }_{1}\times \frac{2\Delta x\times {x}_{n}}{(y{p}_{n}-y{q}_{n}+)} ,\end{eqnarray*}

where

(17) \begin{eqnarray*}y{p}_{n}=rand\times \left( {y}_{n}+rand\times \Delta x \right) ,  y{q}_{n}=rand\times \left( {y}_{n}-rand\times \Delta x \right) ,\end{eqnarray*}

*y*_*n*_ is given in Eq. (15), and

(18) \begin{eqnarray*}{z}_{n+1}={x}_{n}-randn\times \frac{2\Delta x\times {x}_{n}}{({x}_{worst}-{x}_{best}+)} ,\end{eqnarray*}

where *ɛ* is a small number within the range of [0, 0.1], *randn* is a normally distributed random number, *x*_*best*_ and *x*_*worst*_ are the best and worst solutions obtained in the search progress, and Δ*x* is given by

\begin{eqnarray*}\Delta x=rand(1:{N}_{p})\times {|}step{|}, \end{eqnarray*}

\begin{eqnarray*}step= \frac{{x}_{best}-{x}_{r1}^{m}+\delta }{2} , \end{eqnarray*}

\begin{eqnarray*}\delta =2\times rand\times \left( {|} \frac{{x}_{r1}^{m}+{x}_{r2}^{m}+{x}_{r3}^{m}+{x}_{r4}^{m}}{4} {|} \right) , \end{eqnarray*}

where *rand*(1:*N*_*p*_) is a random number with *N*_*p*_ dimensions, *r*_1_, *r*_2_, *r*_3_, and *r*_4_(*r*_1_ ≠ *r*_2_ ≠ *r*_3_ ≠ *r*_4_ ≠ *n*) are different integers randomly chosen from [1, *N*_*p*_]. The parameter *ρ*_1_ in Eq. (16) is incorporated to enhance the search capability and to balance the exploration and exploitation phases in order to explore the promising regions in the search space and eventually converge to the global optimal solution. This parameter is given by

\begin{eqnarray*}{\rho }_{1}=2\times rand\times \alpha -\alpha , \end{eqnarray*}

\begin{eqnarray*}\alpha ={|}\beta \sin \nolimits \left( \frac{3\pi }{2} +\sin \nolimits \left( \beta \frac{3\pi }{2} \right) \right) {|}, \end{eqnarray*}

(19) \begin{eqnarray*}\beta ={\beta }_{min}+({\beta }_{max}-{\beta }_{min})\times { \left( 1-{ \left( \frac{m}{M} \right) }^{3} \right) }^{2},\end{eqnarray*}

where *β*_*min*_ and *β*_*max*_ are 0.2 and 1.2, respectively, *m* is the number of iterations, and *M* is the total number of iterations.

Beside *GSR*, the direction of movement (*DM*) is included to boost the exploitation in the nearby area of *x*_*n*_. *DM* is given by

(20) \begin{eqnarray*}DM=rand\times {\rho }_{2}\times \left( {x}_{best}-{x}_{n} \right) ,\end{eqnarray*}

where *rand* is a random number in [0, 1], and

(21) \begin{eqnarray*}{\rho }_{2}=2\times rand\times \alpha -\alpha .\end{eqnarray*}

As can be seen, *DM* moves *x*_*n*_ in the direction of *x*_*best*_ − *x*_*n*_, which improves the local search tendency.

Now, the position $X{1}_{n}^{m}$ is defined as

(22) \begin{eqnarray*}X{1}_{n}^{m}={x}_{n}^{m}-GSR+DM={x}_{n}^{m}-randn\times {\rho }_{1}\times \frac{2\Delta x\times {x}_{n}^{m}}{(y{p}_{n}^{m}-y{q}_{n}^{m}+)} \nonumber\\\displaystyle  +rand\times {\rho }_{2}\times ({x}_{best}-{x}_{n}^{m}).\end{eqnarray*}

The position $X{2}_{n}^{m}$ is obtained from $X{1}_{n}^{m}$ by replacing *x*_*best*_ with ${x}_{n}^{m}$, that is,

(23) \begin{eqnarray*}X{2}_{n}^{m}={x}_{best}-randn\times {\rho }_{1}\times \frac{2\Delta x\times {x}_{n}^{m}}{(y{p}_{n}^{m}-y{q}_{n}^{m}+)} +rand\times {\rho }_{2}\times ({x}_{r1}^{m}-{x}_{r2}^{m}).\end{eqnarray*}

Notice that Eq. (22) emphasizes the exploration process and Eq. (23) emphasizes the exploitation process. Thus, to enhance both exploration and exploitation phases, the vector of the next iteration ${x}_{n}^{m+1}$ is computed by

(24) \begin{eqnarray*}{x}_{n}^{m+1}={r}_{a}\times \left( {r}_{b}\times X{1}_{n}^{m}+(1-{r}_{b})\times X{2}_{n}^{m} \right) +(1-{r}_{a})\times X{3}_{n}^{m},\end{eqnarray*}

where

(25) \begin{eqnarray*}X{3}_{n}^{m}={X}_{n}^{m}-{\rho }_{1}\times (X{2}_{n}^{m}-X{1}_{n}^{m}),\end{eqnarray*}

where *r*_*b*_ and *r*_*a*_ are two random numbers in [0, 1], and ${X}_{n}^{m}$ is the current position.

#### Local escaping operator

To enhance the performance of GBO in tackling complex problems, the local escaping operator (LEO) is utilized to generate a new vector with a superior performance ${X}_{LEO}^{m}$. The assigning of the solution of the next iteration ${x}_{n}^{m+1}$ to ${X}_{LEO}^{m}$ is based on a probability rate *pr*. The LEO can provide a significant change for the position of ${x}_{n}^{m+1}$ to avoid stagnation in local optimal solutions. Based on a random number, the solution ${X}_{LEO}^{m}$ is either computed by

(26) \begin{eqnarray*}{X}_{LEO}^{m}={X}_{n}^{m+1}+{f}_{1}\times ({u}_{1}\times {x}_{best}-{u}_{2}\times {x}_{k}^{m})+{f}_{2}\times {\rho }_{1}\times ({u}_{3}\times (X{2}_{n}^{m}-X{1}_{n}^{m})\nonumber\\\displaystyle  +{u}_{2}\times ({x}_{r1}^{m}-{x}_{r2}^{m}))/2,\end{eqnarray*}

or computed by

(27) \begin{eqnarray*}{X}_{LEO}^{m}={x}_{best}+{f}_{1}\times ({u}_{1}\times {x}_{best}-{u}_{2}\times {x}_{k}^{m})+{f}_{2}\times {\rho }_{1}\times ({u}_{3}\times (X{2}_{n}^{m}-X{1}_{n}^{m})\nonumber\\\displaystyle  +{u}_{2}\times ({x}_{r1}^{m}-{x}_{r2}^{m}))/2,\end{eqnarray*}

where

\begin{eqnarray*}{x}_{k}^{m}={l}_{2}\times {x}_{p}^{m}+(1-{l}_{2})\times {x}_{rand},{x}_{rand}={X}_{min}+rand(0,1)\times ({X}_{max}-{X}_{min}), \end{eqnarray*}

and

\begin{eqnarray*}{u}_{1}={l}_{1}\times 2\times rand+(1-{l}_{1}),~~~~{u}_{2}={l}_{1}\times rand+(1-{l}_{1}),\nonumber\\\displaystyle {u}_{3}={l}_{1}\times rand+(1-{l}_{1}). \end{eqnarray*}

Here, *f*_1_ is a uniform random number in the range of [ − 1, 1], *f*_2_ is a random number from a normal distribution with mean of 0 and standard deviation of 1. The solution ${x}_{p}^{m}$ is randomly chosen from the population (*p* ∈ [1, 2, …, *N*_*p*_]). *rand* is a random number in the range of [0, 1]. *l*_1_ and *l*_2_ are binary parameters with a value of 0 or 1. The values of *l*_1_ and *l*_2_ are determined based on random numbers in the range of [0, 1], *i.e., μ*_1_ and *μ*_2_. That is, if *μ*_1_ < 0.5, then *l*_1_ = 1, otherwise *l*_1_ = 0. Similarly, if *μ*_2_ < 0.5, then *l*_2_ = 1, otherwise *l*_2_ = 0. Notice that, assigning *u*_1_, *u*_2_, and *u*_3_ to random values promotes the diversity of the population, and enhances the escapement from local optimal solution. The pseudo code of GBO is given in Algorithm 1 .


 
_______________________ 
Algorithm 1 The pseudo code of GBO.__________________________________________________ 
  Step 1. Initialization 
  Assign values for parameters M, ɛ, and pr. 
  Generate an initial population X0 = [x0,1,x0,2,...,x0,D]. 
  Evaluate the objective function value f(X0),n=1,...,Np. 
  Specify the best and worst solutions xmbest and xmworst. 
  Step 2. Main loop 
  while m < M do 
      for n = 1 : Np do 
          for i = 1 : D do 
              Select randomly r1 ⁄= r2 ⁄= r3 ⁄= r4 ⁄= n in the 
                range of [1,Np]. 
                Calculate the position xm+1 
n,i   using (??) 
           end for 
          Local escaping operator 
          if rand < pr then 
              if rand < 0.5 then 
                  Calculate the position xmLEO using (??) 
                else 
                  Calculate the position xmLEO using (??) 
                end if 
              Xm+1n = xmLEO. 
           end if 
          Update the positions xmbest and xmworst. 
       end for 
      m = m + 1. 
  end while 
  Step 3. return xmbest.________________________________________________________________________    


### The proposed design method

The proposed design method involves the appropriate utilizing of GBO to solve the optimization problem given in Eq. (12). Being a metaheuristic technique, GBO gives a different solution $\overline{\mathbf{q}}$ for Eq. (12) at each independent run. As such, our method relies on repetitive execution of GBO on Eq. (12) and choosing the best approximation filter that satisfy the design requirements.

In general, metaheuristic techniques start with a set of random solutions and utilize them to converge to an optimal solution with the course of iterations. Therefore, researchers resort to run a metaheuristic technique on the problem of interest for *T*_2_ times and then choose the best solution among the obtained solutions. As one can expect, the larger *T*_2_ is, the larger is the probability of getting better solution. The price to be paid for a probable increase of accuracy is a significant increase of execution time.

Fortunately, in our method we figured out that all the obtained solutions have quite similar performance since the proposed objective function guides the metaheuristic technique towards the most feasible regions of the search space. This is because our optimization problem is formulated with tuning parameters that can be used to effectively control the error profile over the Nyquist band.

We consider the parameters *γ*, *λ*, *ν*, and *N* as user-defined parameters that can be set according to the application requirements. The parameters need to be defined in our method are *ϵ*_*m*_, *ϵ*_*s*_, *μ*_*m*_, *μ*_*ϕ*_, *N*_*p*_, *M*, and *T*_2_. Both *ϵ*_*m*_ and *ϵ*_*s*_ are set to 0.01. To impose a tighter limit on the magnitude error than the phase error, *μ*_*m*_ and *μ*_*ϕ*_ are set to 0.9 and 0.1 respectively. We put *N*_*p*_ = 50 since it results in the optimal performance of GBO as has been reported in the related literature. The maximum number of iterations for GBO is set to *M* = 500, where we have observed that GBO often converges to its final solution at 400 < *M* < 500 and that adopting more iterations can only increases the execution time without significant improvement. Based on many experiments, we figured out that *T*_2_ = 100 is almost sufficient number of runs to come to the best attainable solution.

In summary, the proposed design method of DFODs is broken into the following steps.

 
_______________________________________________________________________________________________________ 
Algorithm 2 The proposed methodology________________________________________________ 
  Define ϵm, ϵs, μm, μϕ, Np, M, and T2. 
  Input γ, λ, ν, N. 
  Formulate (??). 
  while t2 < T2 do 
      Execute GBO on (??). 
       __q ← Xα. 
       H(ejω,q) = γνHγ(ejω,__q). 
       H(t2) = H(ejω,q). 
       t2 ← t2 + 1. 
  end while 
  qopt ← optimal coefficients among H. 
  return qopt__________________________________________________________________________________________________________    

The computational complexity of the proposed method mainly depends on that of GBO and on *T*_2_. GBO consists of three main processes, namely, initialization, evaluation of the objective function, and updating of positions. The computational complexity of initializing *N*_*p*_ solutions is *O*(*N*_*p*_). The computational complexity of updating positions involves that of searching for the best position (which is *O*(*M* × *N*_*p*_)) and that of calculating the vector for all positions (which is *O*(*M* × *N*_*p*_ × *D*)). Remember that *M* is the maximum number of iterations and *D* is the length of the position vector. From Eq. (2), it can be observed that *D* = 3 × *N* + 2 for *N* odd and *D* = 3 × *N* + 1 for *N* even, where *N* is the filter order. This demonstrates that the computational complexity of GBO is *O*(*N*_*p*_ × (*M* + *M* × *D* + 1)) (Daoud et al., 2023). The computational complexity of searching for **q**_opt_ within **H** is *O*(*T*_2_). Therefore, the computational complexity of the proposed method is *O*(*T*_2_ × (*N*_*p*_ × (*M* + *M* × *D* + 1) + 1)).

## Simulation results

In this section, we present design examples with various combinations of the design parameters *γ*, *λ*, and *ν* to investigate the performance of the proposed method. The performance metrics used in what follows are the relative magnitude-response error (in dB), the absolute phase-response error, and the group delay error. The relative magnitude-response error is given by

(28) \begin{eqnarray*}{e}_{m}(\omega ,\mathbf{q})= \frac{ \left[ H({e}^{j\omega },\mathbf{q})-{\omega }^{\nu } \right] }{{\omega }^{\nu }} .\end{eqnarray*}

The phase-response error is given by

(29) \begin{eqnarray*}{e}_{\phi }(\omega ,\mathbf{q})= \left[ {\phi }_{d}(\omega ,\nu ,\theta )-{\phi }_{H}({e}^{j\omega },\nu ,\theta ) \right] .\end{eqnarray*}

The group delay response is given by

(30) \begin{eqnarray*}{\tau }_{g}=- \frac{d\angle H({e}^{j\omega },\mathbf{q})}{d\omega } \end{eqnarray*}

We also use the maximum relative magnitude and phase errors, namely,

(31) \begin{eqnarray*}{\delta }_{r}=\max \nolimits {|}{e}_{m}(\omega ,\mathbf{q}){|}, \omega \in {\Omega }_{P},\end{eqnarray*}

and

(32) \begin{eqnarray*}{\delta }_{\phi }=\max \nolimits {|}{e}_{\phi }(\omega ,\mathbf{q}){|}, \omega \in {\Omega }_{P}.\end{eqnarray*}

Notice that, Ω_*P*_ is set to [0.05*π*, 0.1*π*] in all design examples since our focus in this paper is to improve the performance over low-frequency bands.

For comparison reasons, we investigate the performance of the proposed method *versus* two benchmarks: real-coded genetic algorithm (RCGA) as the best evolutionary algorithms (Holland, 1992) and particle swarm optimization (PSO) as the best of swarm intelligence-based algorithms (Kennedy & Eberhart, 1995) in terms of accuracy, robustness, and consistency. We thus use these methods to design one-half and one-fourth differentiators that satisfy some design criteria. Three examples are given below, where third-order IIR filters are utilized to meet the given design specifications. To have a reliable comparison, a count of 100 trial independent runs for each single design have been performed, and the poles and zeros have been saved. The simulations have been carried out in MATLAB 9.0 operating on Intel(R) Core(TM), i7-4510U CPU @ 2.6 GHz and 8 GB RAM. The control parameters for the metaheuristics being compared are presented in Table 1.

**Table 1:** Control parameters of the employed metaheuristics.

Parameter	RCGA	PSO	GBO
Population size	50	50	50
Maximum iterations	500	500	500
Crossover function	Two-point	–	–
Mutation rate	0.1	–	–
Selection method	Roulette wheel	–	–
Cognitive constant	–	2.0	–
Social constant	–	2.0	–
Initial velocity	–	0.05	–
Final velocity	–	1	–
Minimum inertia weight	–	0.4	–
Maximum inertia weight	–	0.9	–

### Example 1

**Figure 1:**
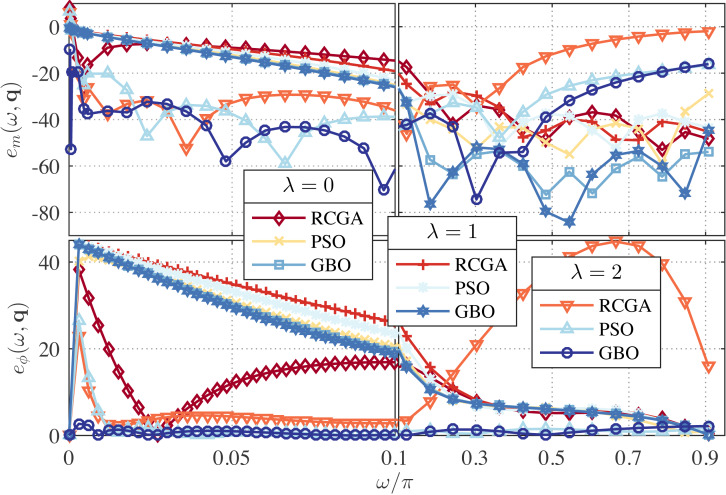
Relative magnitude error (in dB) and phase error (in degree) for the RCGA-, PSO-, and GBO-based one-half DFODs with *γ* = 1.

**Figure 2:**
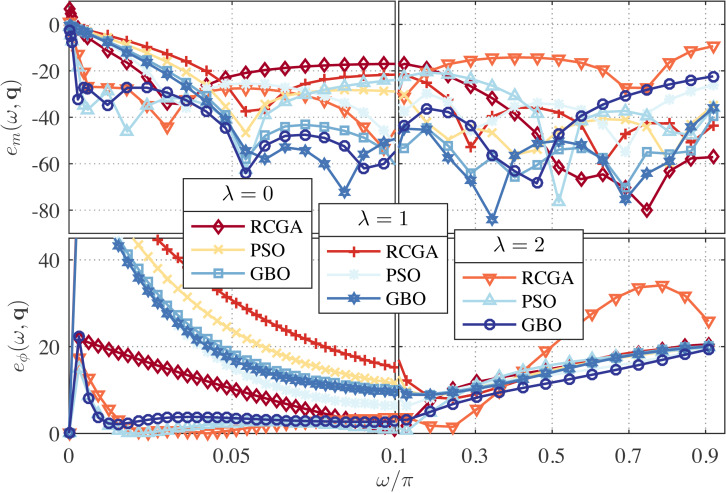
Relative magnitude error (in dB) and phase error (in degree) for the RCGA-, PSO-, and GBO-based one-fourth DFODs with *γ* = 1.

In this example, we set *γ* = 1 and use *λ* as a parameter with the values *λ* = 0, 1, and 2. The zeros, poles and performance metrics of the obtained approximations are presented in Table 2. From this table, the GBO-based approximations outperform the RCGA- and PSO-based ones in terms of *δ*_*r*_ in all cases. This can be clearly observed from Figs. 1 and 2 that depict the respective frequency response errors, where the low-frequency band [0, 0.1*π*] has been enlarged due to the particular significance of the band in this paper.

**Table 2:** Transfer functions of the RCGA, PSO and GBO-based DFODs for *γ* = 1.

*α*	*λ*	Alg.		Zeros			Poles			Performance metrics	
			*k*	*z*_0_	*z*_1_	*z*_2_	*p*_0_	*p*_1_	*p*_2_	*δ*_*r*_(dB)	*δ*_*ϕ*_(°)
1/2	0	RCGA	−0.9999	1.0000	0.2789	0.2048	0.6885	0.0817	0.0605	−9.4	12.03
		PSO	−1.0000	−1.6534	0.1798	0.0818	0.6689	0.1682	−0.1155	−12.22	30.83
		**GBO**	**1.0000**	**−0.5237**	**1.0000**	**0.3207**	**−0.5634**	**0.0255**	**0.6546**	**−12.87**	**29.87**
	1	RCGA	−0.9999	0.9879	0.6124	−0.0222	0.8738	0.3449	−0.2595	−9.73	34.89
		PSO	−0.9996	0.9823	0.4449	0.2873	0.8685	0.1853	0.0404	−11.04	33.26
		**GBO**	**1.0000**	**−0.4828**	**0.3401**	**1.0000**	**0.6638**	**−0.5273**	**0.0430**	**−13.13**	**29.47**
	2	RCGA	−1.0000	0.9533	0.3276	−0.1015	0.8044	−0.4028	0.1198	−24.4	20.69
		PSO	0.9994	0.9885	0.7787	0.2920	0.9309	0.4811	0.1411	−32.71	1.362
		**GBO**	**1.0000**	**0.9954**	**0.5633**	**0.9262**	**0.1819**	**0.8058**	**0.9768**	**−37.10**	**1.43**
1/4	0	RCGA	−0.9991	−0.2980	−0.7835	0.6094	0.3182	−0.7874	−0.3329	−16.96	11.8
		PSO	−1.0000	0.9996	0.0134	0.3990	0.8669	0.2691	−0.0657	−28.89	23.7
		**GBO**	**1.0000**	**0.5479**	**−0.1756**	**1.0000**	**−0.2478**	**0.8977**	**0.3844**	**−44.27**	**18.5**
	1	RCGA	0.9986	0.0693	0.0750	1.0000	−0.0946	0.8236	0.0727	−21.54	30.6
		PSO	0.9999	0.6623	1.0000	0.5791	0.7468	0.2804	0.8938	−25.41	15.1
		**GBO**	**1.0000**	**−0.0891**	**0.5907**	**1.0000**	**−0.1770**	**0.9061**	**0.4385**	**−43.31**	**16.92**
	2	RCGA	−0.9724	0.9400	0.1293	−0.1802	−0.5386	0.1414	0.8774	−15.14	5.34
		PSO	1.0000	0.3875	0.5993	0.9615	0.1175	0.5770	0.9243	−20.65	11.0
		**GBO**	**1.000**	**0.9170**	**0.4559**	**1.0000**	**0.8528**	**0.2561**	**0.9912**	**−36.09**	**8.2**

From Fig. 1, it is also apparent that for *λ* = 2 the low frequency magnitude error is significantly reduced and the phase error is reduced over the entire Nyquist band, but at the cost of large magnitude error in the high frequency bands. Note that the case *γ* = 1 and *λ* = 0 is reduced to the *L*_1_-norm method used in Ali et al. (2020), and the case *γ* = 1 and *λ* = 1 is reduced to the method proposed in Ali et al. (2019). As can be seen from Fig. 1, both cases result in poor low-frequency performance. Of course, tuning *λ* between 1 and 2 can yield many design compromises with error profiles between the *λ* = 1 and *λ* = 2 ones.

### Example 2

**Figure 3:**
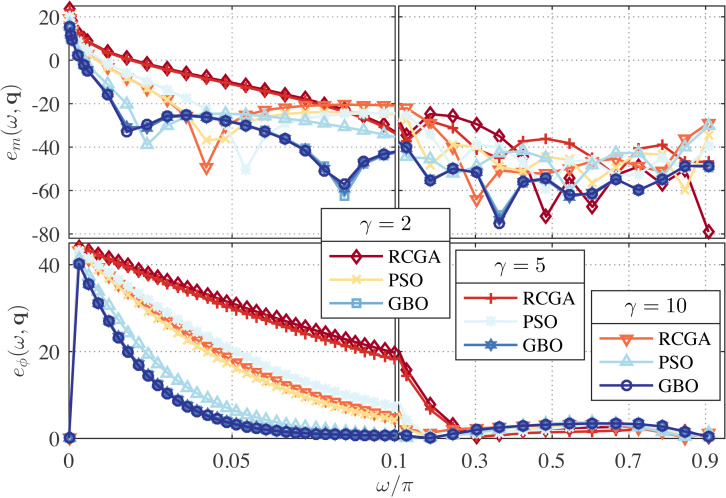
Relative magnitude error (in dB) and phase error (in degree) for the RCGA-, PSO-, and GBO-based one-half DFODs with *λ* = 0.

**Figure 4:**
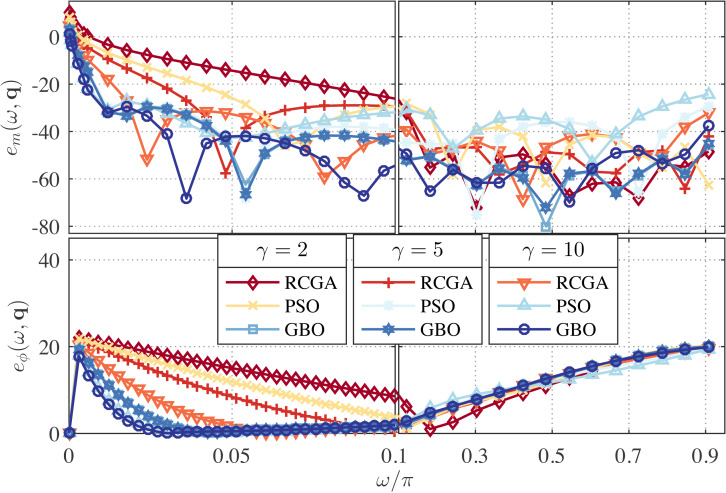
Relative magnitude error (in dB) and phase error (in degree) for the RCGA-, PSO-, and GBO-based one-fourth DFODs with *λ* = 0.

In this example, we set *λ* = 0 and use *γ* as a parameter with the values *γ* = 2, 5, and 10. The zeros, poles, and performance metrics of the obtained approximations are given in Table 3. The corresponding magnitude- and phase-error curves are depicted in Figs. 3 and 4. As can be seen from Table 3, the maximum magnitude- and phase-response errors, *δ*_*r*_ and *δ*_*ϕ*_, of the GBO-based one-half DFODs are −28.46 dB and 3.39° respectively, which are less than those of the RCGA- and PSO-based counterparts. The maximum magnitude- and phase-response errors of the GBO-based one-fourth DFODs are ≈ − 44.7 dB and 7.76°, which are significantly less than the RCGA- and PSO-based counterparts in most cases.

**Table 3:** Transfer functions of the RCGA, PSO and GBO-based DFODs for *λ* = 0.

*α*	*γ*	Alg.		Zeros			Poles			Performance metrics	
			*k*	*z*_0_	*z*_1_	*z*_2_	*p*_0_	*p*_1_	*p*_2_	*δ*_*r*_(dB)	*δ*_*ϕ*_(°)
1/2	2	RCGA	1.0829	−0.3724	−0.4129	0.5810	−0.3154	0.1066	−0.5107	−10.43	31.05
		PSO	1.0614	0.0479	0.2112	0.7974	0.3850	−0.1976	0.3100	−24.87	16.57
		**GBO**	**1.0544**	**0.9097**	**0.4383**	**−0.4159**	**0.1087**	**0.7254**	**−0.4778**	**−28.46**	**3.39**
	5	RCGA	1.0900	−1.0000	−0.6029	0.6107	−1.0000	0.1431	−0.6451	−11.15	29.92
		PSO	1.0657	−0.0504	0.7613	−0.1606	0.3583	−0.3049	−0.0560	−24.32	20.22
		**GBO**	**1.0545**	**0.4403**	**0.9096**	**−0.4116**	**−0.4743**	**0.1108**	**0.7263**	**−28.46**	**3.39**
	10	RCGA	1.0521	0.1507	0.7805	0.1053	0.2701	−0.1474	0.3472	−20.83	17.94
		PSO	1.0515	0.8891	0.1284	0.6356	0.4764	−0.1272	0.7328	−24.68	6.55
		**GBO**	**1.0543**	**0.9108**	**0.4409**	**−0.4129**	**−0.4754**	**0.7275**	**0.1107**	**−28.46**	**3.39**
1/4	2	RCGA	1.0531	−0.2965	0.4930	0.0136	0.2964	−0.3503	0.0256	−14.38	15.01
		PSO	1.0354	−0.1631	0.5943	0.0144	0.2877	−0.2937	0.1814	−25.86	11.81
		**GBO**	**1.0258**	**0.4857**	**−0.2484**	**0.9093**	**−0.3071**	**0.3227**	**0.8418**	**−41.66**	**7.76**
	5	RCGA	1.0309	0.7164	−0.0640	−0.2269	0.5423	−0.1654	−0.2299	−28.93	8.38
		PSO	1.0260	0.2552	0.4041	0.9036	0.1517	0.8284	0.3007	−37.93	8.26
		**GBO**	**1.0258**	**0.4884**	**0.9095**	**−0.2417**	**0.8424**	**−0.3015**	**0.3262**	**−41.66**	**7.76**
	10	RCGA	1.0268	0.1996	0.8573	0.2389	0.1780	0.7566	0.0796	−32.72	7.5
		PSO	1.0133	0.9363	0.4093	0.5199	0.3606	0.8871	0.3413	−30.51	9.0
		**GBO**	**1.0243**	**0.9407**	**0.6319**	**−0.0076**	**0.4956**	**0.8951**	**−0.1157**	**−41.79**	**7.76**

From Fig. 3, it is apparent that if *γ* ≥ 2 the magnitude- and phase-response errors are significantly reduced in the low frequency bands as compared to *γ* = 1 (*see* Fig. 1). However, increasing *γ* up beyond 2 results in an insignificant change of error curves. For the one-fourth approximations, it is observed from Fig. 4 that the *γ* = 2 approximation performs better than the *γ* = 5 and 10 counterparts at the low frequency and better than the *γ* = 1 counterpart as well, meanwhile its performance at mid- and high-frequency bands is commendable. This means that the value of *γ* can be tuned to attain a favorable wideband approximation. Regarding the phase response, no significant change in the error curves is observed for both one-half and one-fourth approximations.

### Example 3

**Figure 5:**
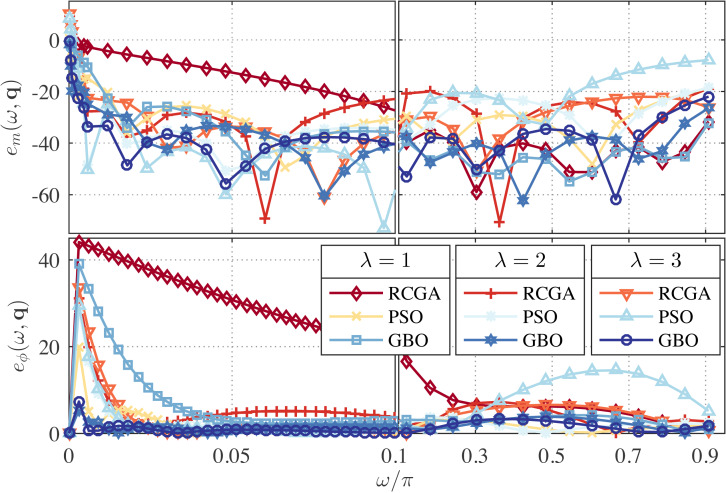
Relative magnitude error (in dB) and phase error (in degree) for the RCGA-, PSO-, and GBO-based one-half DFODs with *γ* = 5.

**Figure 6:**
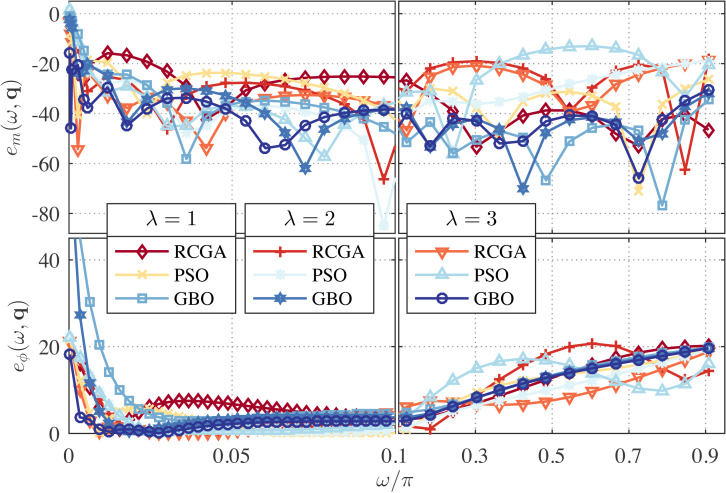
Relative magnitude error (in dB) and phase error (in degree) for the RCGA-, PSO-, and GBO-based one-fourth DFODs with *γ* = 5.

In this example, we set *γ* = 5 and use *λ* as a parameter. The approximations for *λ* = 1, 2, and 3 are obtained and summarized in Table 4. As seen from this table, the GBO-based one-half DFODs achieve less maximum magnitude and phase errors, *δ*_*r*_ and *δ*_*ϕ*_, than the RCGA- and PSO-based counterparts. The GBO-based one-fourth approximations achieve better results in most cases. The corresponding frequency error curves for the one-half and one-fourth approximations are depicted in Figs. 5 and 6, respectively. These figures demonstrate that the GBO-based approximations exhibit better or competitive performance over the band [0, 0.1], while consistently performing better up to the Nyquist.

**Table 4:** Transfer functions of the RCGA, PSO and GBO-based DFODs for *γ* = 5.

*α*	*λ*	Alg.		Zeros			Poles			Performance metrics	
			*k*	*z*_0_	*z*_1_	*z*_2_	*p*_0_	*p*_1_	*p*_2_	*δ*_*r*_(dB)	*δ*_*ϕ*_(°)
1/2	1	RCGA	0.9994	0.2084	1.0000	−0.0871	−0.1751	−0.0013	0.6228	−12.71	30.71
		PSO	1.0288	0.0867	0.9825	0.6807	0.9024	0.2715	0.0311	−29.8	3.47
		**GBO**	**1.0402**	**0.7693**	**0.0749**	**1.0000**	**0.9281**	**−0.1599**	**0.4783**	**−35.37**	**3.23**
	2	RCGA	1.0288	0.9690	0.5722	−0.3892	−0.0633	−0.2602	0.8832	−19.74	5.197
		PSO	1.0492	0.7866	0.9742	0.6263	0.7092	0.2360	0.9119	−23.16	3.8
		**GBO**	**1.0438**	**0.8723**	**0.9917**	**0.3268**	**0.6734**	**0.9599**	**−0.0229**	**−33.78**	**2.38**
	3	RCGA	1.0812	0.9616	0.6146	0.0273	0.8582	−0.1703	0.2836	−29.26	5.3
		PSO	0.9280	0.7513	−0.3444	0.9725	0.2958	−0.1469	0.9061	−20.31	4.51
		**GBO**	**1.0378**	**0.4792**	**0.9088**	**0.9918**	**0.0772**	**0.9684**	**0.7680**	**−36.99**	**3.02**
1/4	1	RCGA	1.0261	−0.1873	0.4610	0.9396	−0.2773	0.8512	0.3493	−25.17	6.88
		PSO	1.0206	0.3907	0.9631	−0.1721	−0.0984	0.0836	0.9092	−23.8	9.4
		**GBO**	**1.0185**	**0.7969**	**1.0000**	**0.1514**	**0.9752**	**0.6772**	**−0.0036**	**−34.82**	**8.17**
	2	RCGA	0.9945	0.9423	−0.7107	0.0796	−0.3464	0.8808	−0.5519	−19.01	8.58
		PSO	0.9804	0.5219	0.9353	0.3473	0.2746	0.8784	0.4100	−34.68	6.47
		**GBO**	**1.0198**	**0.2675**	**1.0000**	**0.8696**	**0.9881**	**0.0813**	**0.7741**	**−33.65**	**8.17**
	3	RCGA	1.0202	0.9746	0.0666	0.6874	0.4133	0.9483	0.1453	−20.77	7.55
		PSO	1.0960	0.3872	0.9387	−0.5315	0.8863	−0.2009	−0.2106	−26.93	14.95
		**GBO**	**1.0207**	**0.3080**	**0.8723**	**0.9920**	**0.1152**	**0.7871**	**0.9811**	**−38.37**	**8.17**

**Notes.**

The outcomes of the proposed method are shown in bold.

Moreover, it is observed from this Fig. 5 that increasing *λ* does not increase the error at the high frequency bands as fast as happened for *γ* = 1 (see Fig. 1). Yet, the phase response is improved over the entire Nyquist band as *λ* goes large. For the one-fourth approximations, it is clear that increasing *λ* improves the low frequency performance, and increases the error at high frequencies but slower than the *ν* = 1/2 case.

The overall performance of GBO is compared with those of RCGA and PSO based on an average-, best- and worst-case analysis over the 100 independent runs. The outcomes of the conducted study are summarized in Table 5, where the minimum, maximum and average values of *δ*_*r*_ and *δ*_*ϕ*_ along with standard deviations are given. Based on the results shown in Table 5, GBO achieves better average values of all *δ*_*r*_ and *δ*_*ϕ*_ statistics for both one-half and one-fourth approximations, which indicates a steady superiority of GBO over RCGA and PSO in terms of the considered problem. In addition, the standard deviation of the GBO-based approximations are the smallest in most cases, demonstrating a more stable performance and robustness of GBO in the considered problem.

The performance of the proposed approach and that of the benchmark approaches are statistically tested over the 100 independent runs for each design case using the Wilcoxon rank-sum test for the metric *δ*_*r*_. Pair-wise combinations of *n*_1_ outcomes from those of the benchmark algorithm and *n*_2_ outcomes from those of the proposed approach are used. The corresponding sum of ranks, *W*_1_ and *W*_2_, are compared to the critical values given in the Wilcoxon rank-sum tables (Montgomery & Runger, 2011) to decide whether the null hypothesis is accepted or at what level of significance, *a*, it is rejected. The confidence level of the difference between the outcomes, *n*_1_ and *n*_2_, is *CL* = (1 − *a*) × 100%. The results of this statistical test are summarized in Table 6. Apparently, the null hypothesis is rejected with high confidence levels in most design outcomes, which confirms that GBO is consistently superior over RCGA and PSO in the considered problem in terms of *δ*_*r*_.

**Table 5:** Performance metrics of the RCGA, PSO and GBO-based DFODs.

			*α* = 1/2								*α* = 1/4							
			*δ*_*r*_				*δ*_*ϕ*_				*δ*_*r*_				*δ*_*ϕ*_			
			min.	max.	ave.	std.	min.	max.	ave.	std.	min.	max.	ave.	std.	min.	max.	ave.	std.
*γ*	*λ*	Alg.	(dB)	(dB)	(dB)						(dB)	(dB)	(dB)		(°)	(°)	(°)	
1	0	RCGA	−9.4	−1.11	−5.47	2.63	12.03	29.96	20.66	5.36	−16.96	−7.01	−11.62	2.79	10.14	25.99	17.28	4.30
		PSO	−12.22	−4.22	−8.43	2.31	12.52	30.83	21.42	5.14	−28.89	−18.15	−23.17	3.21	10.03	25.82	17.17	4.59
		**GBO**	**−12.87**	**−10.02**	**−11.39**	**0.81**	**23.01**	**29.94**	**26.73**	**2.29**	**−44.27**	**−36.03**	**−39.74**	**2.35**	**10.05**	**19.88**	**15.19**	**2.64**
	1	RCGA	−9.73	−1.09	−5.38	2.35	23.21	34.89	28.46	3.35	−21.54	−9.01	−14.54	3.31	13.01	20.0	16.90	2.09
		PSO	−11.04	−4.03	−7.37	1.97	23.11	33.95	27.45	3.25	−25.41	−15.06	−20.14	2.81	13.06	29.96	21.71	4.84
		**GBO**	**−13.13**	**−9.03**	**−11.18**	**1.19**	**25.09**	**29.93**	**27.35**	**1.33**	**−43.31**	**−35.00**	**−39.09**	**2.41**	**13.01**	**20.0**	**16.90**	**2.09**
	2	RCGA	−24.4	15.05	−19.69	2.45	1.31	21.76	11.25	6.79	−15.14	−6.10	−10.52	2.76	4.16	12.94	8.02	2.71
		PSO	−32.71	−20.03	−26.17	3.48	1.36	21.35	10.93	5.35	−20.65	−12.04	−16.43	2.49	12.04	20.65	16.43	2.49
		**GBO**	**−37.1**	**−28.06**	**−32.95**	**2.50**	**1.34**	**6.91**	**4.36**	**1.64**	**−36.09**	**−29.05**	**−32.85**	**1.83**	**4.24**	**10.91**	**7.43**	**2.01**
2	0	RCGA	−10.43	−2.11	−5.99	2.39	12.40	31.97	22.64	5.73	−14.38	−7.07	−10.67	2.02	8.04	16.88	11.69	2.31
		PSO	−24.87	−17.12	−21.17	2.21	8.03	22.97	15.72	4.40	−25.86	−14.08	−20.04	3.67	8.07	16.96	12.11	2.44
		**GBO**	**−28.46**	**−23.02**	**−25.58**	**1.75**	**3.11**	**12.97**	**7.94**	**3.02**	**−41.66**	**−34.12**	**−38.20**	**2.03**	**7.02**	**14.55**	**10.37**	**1.92**
5		RCGA	−11.15	−4.05	−7.64	2.09	12.20	30.94	21.95	5.55	−28.93	−15.00	−22.39	3.91	6.07	10.96	8.50	1.53
		PSO	−24.32	−17.18	−20.16	2.08	12.27	30.96	20.91	5.47	−37.93	−25.09	−31.95	3.58	6.02	10.91	8.42	1.40
		**GBO**	**−28.46**	**−23.01**	**−25.66**	**1.62**	**3.10**	**12.81**	**7.91**	**2.52**	**−41.66**	**−34.23**	**−38.09**	**2.15**	**6.05**	**11.0**	**8.28**	**1.48**
10		RCGA	−20.83	−7.097	−13.97	4.29	5.31	20.93	12.94	4.52	−32.72	−6.10	−10.69	3.52	6.09	10.91	8.40	1.45
		PSO	−24.68	−11.03	−17.95	4.07	5.36	20.92	12.30	4.39	−30.51	−12.04	−16.53	2.83	6.03	10.99	8.66	1.44
		**GBO**	**−28.46**	**−23.07**	**−26.15**	**1.37**	**3.14**	**13.81**	**8.07**	**3.27**	**−41.79**	**−29.05**	**−32.91**	**2.01**	**6.11**	**10.98**	**8.40**	**1.37**
5	1	RCGA	−12.7	−6.0	−9.6	2.65	5.2	30.7	17.1	7.46	−25.2	−15.3	−20.0	2.80	4.01	9.92	7.08	1.69
		PSO	−29.8	−16.1	−23.0	4.19	3.2	14.7	8.7	3.48	−23.8	−15.4	−19.8	2.45	4.19	9.94	7.23	1.72
		**GBO**	**−35.4**	**−30.0**	**−32.9**	**1.47**	**3.1**	**10.0**	**6.5**	**2.0**	**−34.8**	**−29.0**	**−31.5**	**1.40**	**4.0**	**10.0**	**6.9**	**1.73**
	2	RCGA	−19.7	−10.0	−14.8	2.97	4.04	9.92	7.05	1.59	−19.0	−10.1	−14.7	2.63	4.0	10.0	7.1	1.78
		PSO	−23.2	−16.2	−19.8	2.0	3.01	7.98	5.32	1.52	−34.7	−20.3	−27.2	3.85	4.1	10.0	7.2	1.81
		**GBO**	**−33.8**	**−26.1**	**−29.8**	**2.21**	**2.38**	**5.97**	**4.17**	**1.16**	**−33.7**	**−28.2**	**−31.0**	**1.71**	**4.0**	**10**	**6.9**	**1.76**
	3	RCGA	−29.3	−15.1	−22.4	4.22	4.02	9.89	7.02	1.90	−20.8	−10.0	−15.5	2.98	7.0	15	10.9	2.14
		PSO	−20.3	−15.1	−17.9	1.52	3.08	7.95	5.34	1.49	−26.9	−14.0	−21.2	3.92	7.1	15	11.1	2.58
		**GBO**	**−37.0**	**−31.1**	**−34.2**	**1.62**	**3.02**	**7.98**	**5.02**	**1.25**	**−38.4**	**−34.0**	**−36.4**	**1.31**	**7.0**	**11.9**	**9.4**	**1.41**

**Notes.**

The outcomes of the proposed method are shown in bold.

**Table 6:** Wilcoxon rank-sum test over the 100 independent runs.

			Wilcoxon rank-sum test									
					Critical value		one-half order (*α* = 1/2)			one-fourth order (*α* = 1/4)		
*γ*	*λ*	Benchmark algorithm	*n*_1_	*n*_2_	0.05	0.01	*W*_1_	*W*_2_	reject(CL)	*W*_1_	*W*_2_	reject(CL)
1	0	RCGA	7	11	44	38	28	143	reject(99%)	28	143	reject(99%)
			10	12	85	76	60	193	reject(99%)	55	198	reject(99%)
		PSO	7	11	44	38	40	131	reject(95%)	31	140	reject(99%)
			10	12	85	76	79	174	reject(95%)	60	193	reject(99%)
	1	RCGA	7	11	44	38	32	139	reject(99%)	33	138	reject(99%)
			10	12	85	76	61	192	reject(99%)	64	189	reject(99%)
		PSO	7	11	44	38	37	134	reject(99%)	35	136	reject(99%)
			10	12	85	76	79	174	reject(95%)	71	182	reject(99%)
	2	RCGA	7	11	44	38	37	134	reject(99%)	35	136	reject(99%)
			10	12	85	76	56	197	reject(99%)	71	182	reject(99%)
		PSO	7	11	44	38	35	136	reject(99%)	37	134	reject(99%)
			10	12	85	76	68	185	reject(99%)	73	180	reject(99%)
2	0	RCGA	7	11	44	38	28	143	reject(99%)	28	143	reject(99%)
			10	12	85	76	55	198	reject(99%)	55	198	reject(99%)
		PSO	7	11	44	38	43	128	reject(95%)	31	140	reject(99%)
			10	12	85	76	77	176	reject(95%)	66	187	reject(99%)
5		RCGA	7	11	44	38	32	139	reject(99%)	37	134	reject(99%)
			10	12	85	76	61	192	reject(99%)	71	182	reject(99%)
		PSO	7	11	44	38	40	131	reject(95%)	43	128	reject(95%)
			10	12	85	76	68	185	reject(99%)	79	174	reject(95%)
10		RCGA	7	11	44	38	33	138	reject(99%)	33	138	reject(99%)
			10	12	85	76	56	197	reject(99%)	71	182	reject(99%)
		PSO	7	11	44	38	37	134	reject(99%)	37	134	reject(99%)
			10	12	85	76	71	182	reject(99%)	79	174	reject(95%)
5	1	RCGA	7	11	44	38	32	139	reject(99%)	31	140	reject(99%)
			10	12	85	76	61	192	reject(99%)	66	187	reject(99%)
		PSO	7	11	44	38	35	136	reject(99%)	29	142	reject(99%)
			10	12	85	76	71	182	reject(99%)	61	192	reject(99%)
	2	RCGA	7	11	44	38	28	143	reject(99%)	28	143	reject(99%)
			10	12	85	76	55	198	reject(99%)	55	198	reject(99%)
		PSO	7	11	44	38	38	133	reject(99%)	47	124	accept
			10	12	85	76	67	186	reject(99%)	83	170	reject(95%)
	3	RCGA	7	11	44	38	40	131	reject(95%)	29	142	reject(99%)
			10	12	85	76	71	182	reject(99%)	64	189	reject(99%)
		PSO	7	11	44	38	37	134	reject(99%)	33	138	reject(99%)
			10	12	85	76	68	185	reject(99%)	71	182	reject(99%)

## Comparisons with existing DFODs

In this section, we further investigate the effectiveness of the proposed approach-based designs by conducting a comparative study with the existing designs reported in the literature in terms of frequency response as well as QRS detection.

### Frequency response

In this subsection, the comparisons are conducted in terms of magnitude, phase, and group-delay errors.

For *ν* = 1/2, the proposed approximations of *γ* = 4 with *λ* = 0.8 and *λ* = 1.2 are compared with the fifth-order DFOD obtained by applying the CFE to g_0.6_AE (*z*^−1^) = 2(1 − *z*^−1^)/(9/5 + 1/5*z*^−1^) (Rajasekhar & Krishna, 2020), the ones computed by Ant-lion optimizer (ALO) (Nayak et al., 2019) and by *L*_1_-norm with Multi-verse optimizer (MVO) (Ali et al., 2020). The proposed DFODs are given by

(33) \begin{eqnarray*}{H}_{1}(z)=1.0393 \frac{(z-0.4628)(z-0.9907)(z-0.9022)}{(z-0.0663)(z-0.7559)(z-0.9653)} ,\end{eqnarray*}

and

(34) \begin{eqnarray*}{H}_{2}(z)=1.0446 \frac{(z-0.2407)(z-0.9920)(z-0.8430)}{(z-0.9521)(z-0.6103)(z+0.0711)} .\end{eqnarray*}

**Figure 7:**
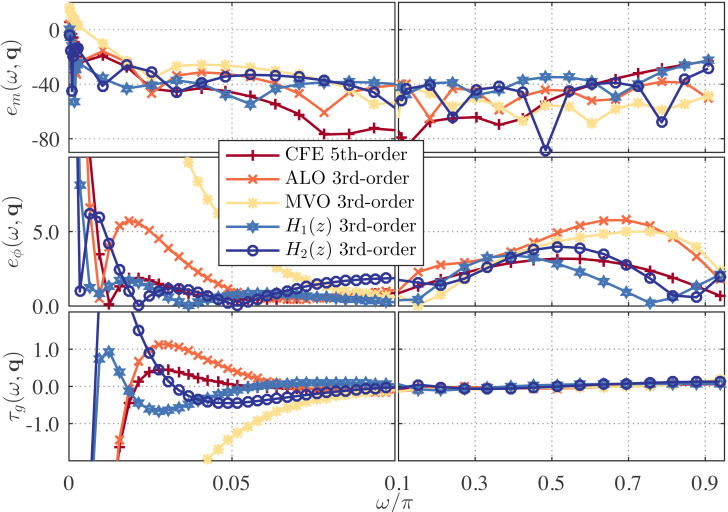
Frequency response error for the one-half differentiators proposed in recent literature, *i.e.,* CFE (Rajasekhar & Krishna, 2020), ALO (Nayak et al., 2019), and MVO (Ali et al., 2020) based DFODs, and for the proposed ones, *H*_1_(*z*) and *H*_2_(*z*).

The error curves are shown in Fig. 7. This figure demonstrates that *H*_1_(*z*) achieves better magnitude accuracy than existing DFODs (including the one by Rajasekhar & Krishna (2020), though it has a higher degree) towards *ω* = 0.0 rad/s, and it maintains a comparable accuracy over the remaining frequency bands. *H*_2_(*z*) performs even better than *H*_1_(*z*) at low frequency, but its magnitude accuracy is worse over high frequencies. In terms of phase and group delay responses, our DFODs perform either better or at least comparable to the others over the entire Nyquist band.

The magnitude errors of the DFODs by Rajasekhar & Krishna (2020), Nayak et al. (2019) and Ali et al. (2020) at low frequency are 5.79, 3.96, and 15.98 dB, respectively. On the other hand, those of our DFODs, *H*_1_(*z*) and *H*_2_(*z*), are −4.04 and 0.78 dB, respectively. Thus, *H*_1_(*z*) achieves a relative error reduction of ≈60% at low frequency as compared to the one by Nayak et al. (2019).

For *ν* = 1/4, the proposed approximations of *γ* = 5 with *λ* = 3 and *γ* = 2 with *λ* = 0 are compared with the third-order DFODs obtained by nelder-mead simplex algorithm (NMSA) (Rana et al., 2016), flower pollination algorithm (FPA) (Mahata et al., 2018b) and by *L*_1_-norm with Multi-verse optimizer (MVO) (Ali et al., 2020). The proposed DFODs are given by

(35) \begin{eqnarray*}{H}_{3}(z)=1.0207 \frac{(z-0.3080)(z-0.8723)(z-0.9920)}{(z-0.1152)(z-0.7871)(z-0.9811)} ,\end{eqnarray*}

and

(36) \begin{eqnarray*}{H}_{4}(z)=1.0258 \frac{(z-0.4857)(z+0.2484)(z-0.9093)}{(z+0.3071)(z-0.3227)(z0.8418)} .\end{eqnarray*}

**Figure 8:**
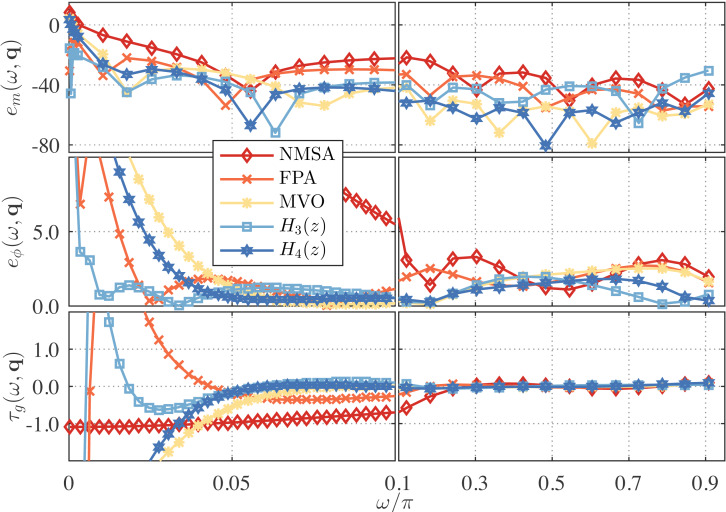
Frequency response error for the one-fourth differentiators proposed in recent literature, *i.e.,* NMSA (Rana et al., 2016), FPA (Mahata et al., 2018b), and MVO (Ali et al., 2020) based DFODs, and for the proposed ones, *H*_3_(*z*) and *H*_4_(*z*).

The error curves are shown in Fig. 8. As in the *ν* = 1/2 case, the proposed approximations exhibit better performance at low frequencies below *ω* = 0.1*π* rad/s, while having competitive performance onwards.

In summary, one may observe the flexibility of our method to attain various design compromises that can be chosen to fit a specific application.

### Application examples

Although simulation can be insightful, but the true verification of the performance of DFODs can only be done by using them in real applications. To do so, we experimentally verify the performance of our design method for the ECG QRS detection and compare the proposed method with state-of-the-art ones.

**Figure 9:**
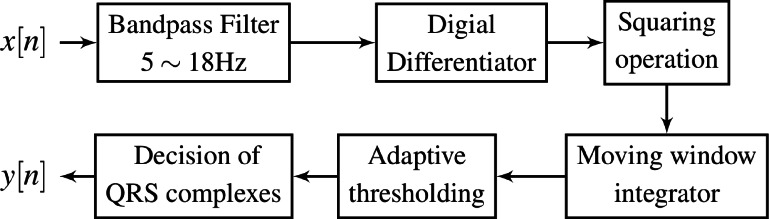
Pan-Tompkins QRS detection method.

In the conducted experiments, we used the Pan-Tompkins algorithm shown in Fig. 9 where the differentiator block is implemented by one of the compared DFODs in the previous section with *ν* = 1/2. Four ECG datasets with various artifacts and pathology are tested, *i.e.,* MIT/BIH Arrhythmia (MITDB), MIT/BIH Supraventricular Arrhythmia (SVDB), MIT/BIH ST Change database (STDB), and T-Wave Alternans Challenge (TWADB) databases. It is observed that the associated signals have frequency components in the band [0.02*π*, 0.2*π*]. A bandpass filter is accordingly utilized to filter out the noise contaminated these signals before differentiation. The performance of *H*_2_(*z*) is compared to those of the third-order DFODs by Nayak et al. (2019) and Ali et al. (2020) and the fifth-order DFOD by Rajasekhar (2022).

**Figure 10:**
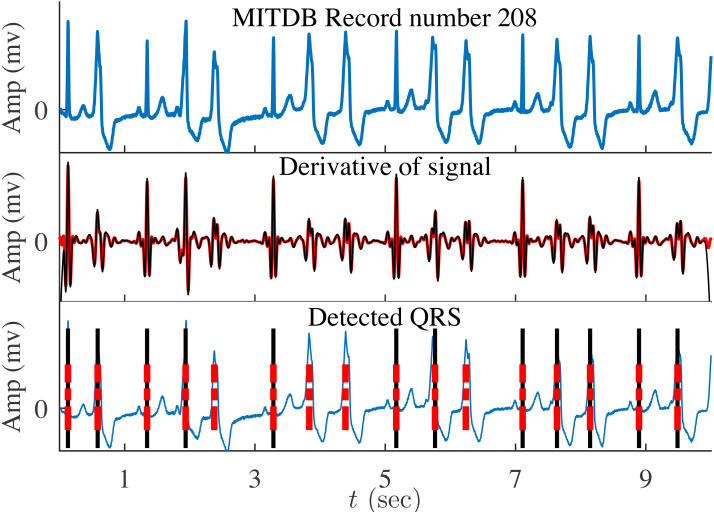
MITDB Record number 208, derivative of signal and detected QRS by *H*_2_(*z*) (red) and by (Nayak et al., 2019)’s model (black).

Figure 10 illustrates the ECG signal for MIT record number 208 along with the output of *H*_2_(*z*) (red) and that of Nayak et al. (2019)’s model(black). Clearly, the number of the undetected QRS is larger in the case of the DFOD due to Nayak et al. (2019). Table 7 presents the comparison in terms of *F*_*N*_ (false negative), *F*_*P*_ (false positive), and *T*_*P*_ (true positive), which correspond to the total number of undetected, wrongly detected, and truly detected heart beats, respectively. These performance indices (*T*_*P*_, *F*_*P*_, and *F*_*N*_) are evaluated with respect to the reference annotations available in PhysioNet. As can be observed from the table, the performance of the proposed approximation is close to that of the fifth-order model by Rajasekhar (2022).

**Table 7:** Comparison of QRS detection.

Database	Model	*F*_*p*_	*F*_*N*_	*F*_*P*_ + *F*_*N*_	*T*_*P*_
MITDB	Nayak et al. (2019)	754	1,188	1,942	107,552
(109494)	Ali et al. (2020)	794	301	1,095	108,399
	Rajasekhar (2022)	767	299	1,066	108,428
	*H*_2_(*z*)	**775**	**294**	**1,069**	**108,425**
SVDB	Nayak et al. (2019)	458	2,647	3,105	181,477
(184582)	Ali et al. (2020)	458	2,645	3,103	181,479
	Rajasekhar (2022)	458	2,647	3,105	181,477
	*H*_2_(*z*)	**454**	**2,651**	**3,105**	**181,477**
STDB	Nayak et al. (2019)	344	2,973	3,317	72,858
(76175)	Ali et al. (2020)	367	2,947	3,314	72,861
	Rajasekhar (2022)	349	2,966	3,315	72,860
	*H*_2_(*z*)	**349**	**2,966**	**3,315**	**72,860**
TWADB	Nayak et al. (2019)	223	1,610	1,833	17,160
(18993)	Ali et al. (2020)	218	120	338	18,655
	Rajasekhar (2022)	200	37	237	18,756
	*H*_2_(*z*)	**201**	**97**	**298**	**18,695**

A consideration is worth mentioning at this point concerning the implementation of our DFODs. Namely, the performance of the proposed approximations can be degraded due to the finite-word-length effects inherent in the digital implementation, including overflow, quantization noise, and round-off and truncation errors. Since such a problem is beyond the scope of this paper, further study can be devoted to the implementation of high-accuracy DFODs on a finite word-length processor.

Finally, Table 8 summarizes the advantages and disadvantages of existing design methods for DFODs along with those of our proposed design method.

**Table 8:** Comparison of existing design methods of DFOD.

Method	Advantages	Disadvantages
Discretization-based	Straightforward	Poor accuracy (narrow band approximations)
	Efficient	No flexibility in choosing frequency band of interest
Gradient-based	Good accuracy (wideband approximations)	Iterative
		Always leads to sub-optimal solution
	Has flexibility to choose mid frequency band of interest	Poor accuracy at low frequencies
Meta-heuristic-based	Good accuracy (wideband approximations)	Iterative
		Frequently stuck in local optima
	Has flexibility to choose mid frequency band of interest	Poor accuracy at low frequencies
Proposed	Good accuracy (wideband approximations)	Iterative
		Slightly less accuracy at high frequencies
	Has flexibility to choose low or mid frequency band of interest	
	Better escapement from local optima	

**Notes.**

The outcomes of the proposed method are shown in bold.

## Conclusions

In this paper, we have proposed a new design method for digital fractional order differentiators (DFODs) with improved accuracy at low frequencies. The new design method is based on a weighted *L*_1_-norm objective function and a recently-proposed metaheuristic algorithm, namely, the gradient-based optimizer (GBO). The proposed objective function includes two tuning parameters that control the frequency error profile such that the low-frequency accuracy can be considerably improved without calling for higher filter order. The utilized metaheuristic algorithm combines the population-based mechanism with a search engine based on the gradient-based Newton’s method to explore the feasible regions in the search space. Based on extensive simulation experiments, it has been confirmed that the proposed method consistently outperforms the well-known metaheuristic approaches, the real-coded genetic algorithm (RCGA) and particle swarm optimization (PSO) in terms of the design of DFODs. The proposed method has been observed to enable the designer to attain accurate wideband low-order DFODs with improved accuracy at low frequencies that compare favorably to existing approximations counterparts. Moreover, the proposed DFODs show promising performance in low-frequency applications, *e.g.*, ECG QRS complexes detection. Future work can be conducted to investigate our design method based approximations in other applications, *e.g.*, signal denoising and control systems.

## Supplemental Information

10.7717/peerj-cs.2341/supp-1 Supplemental Information 1 Code.

10.7717/peerj-cs.2341/supp-2 Supplemental Information 2 The outcomes of the 100 independent runs for the design example number 1 The outcomes are written in three sheets, each for a metaheuristic algorithm among (RCGA, PSO, and GBO). Since the design example involves three design cases, the outcomes within each sheet are given in 12 columns, namely (A-B-C-D for case 1, F-G-H-I for case 2, and K-L-M-N for case 3). The first two columns for each case (e.g., A and B) are corresponding to alpha=1/2, and the last two columns (e.g., C and D) are corresponding to alpha=1/4. The first column contains the maximum relative magnitude error (delta_r), and the second column contains the maximum relative phase error (delta_phi).

10.7717/peerj-cs.2341/supp-3 Supplemental Information 3 The outcomes of the 100 independent runs for the design example number 2 The outcomes are written in three sheets, each for a metaheuristic algorithm among (RCGA, PSO, and GBO). Since the design example involves three design cases, the outcomes within each sheet are given in 12 columns, namely (A-B-C-D for case 1, F-G-H-I for case 2, and K-L-M-N for case 3). The first two columns for each case (e.g., A and B) are corresponding to alpha=1/2, and the last two columns (e.g., C and D) are corresponding to alpha=1/4. The first column contains the maximum relative magnitude error (delta_r), and the second column contains the maximum relative phase error (delta_phi).

10.7717/peerj-cs.2341/supp-4 Supplemental Information 4 The outcomes of the 100 independent runs for the design example number 3 The outcomes are written in three sheets, each for a metaheuristic algorithm among (RCGA, PSO, and GBO). Since the design example involves three design cases, the outcomes within each sheet are given in 12 columns, namely (A-B-C-D for case 1, F-G-H-I for case 2, and K-L-M-N for case 3). The first two columns for each case (e.g., A and B) are corresponding to alpha=1/2, and the last two columns (e.g., C and D) are corresponding to alpha=1/4. The first column contains the maximum relative magnitude error (delta_r), and the second column contains the maximum relative phase error (delta_phi).

## Additional Information and Declarations

### Data Availability

The following information was supplied regarding data availability: The code for designing approximations and raw data are available in the Supplemental Files. The ECG datasets are available at PhysioNet: https://physionet.org/content/mitdb/1.0.0.
